# Intranasal administration of the chemotherapeutic perillyl alcohol results in selective delivery to the cerebrospinal fluid in rats

**DOI:** 10.1038/s41598-021-85293-4

**Published:** 2021-03-18

**Authors:** Geetika Nehra, Shannon Andrews, Joan Rettig, Michael N. Gould, Jill D. Haag, Steven P. Howard, Robert G. Thorne

**Affiliations:** 1 Pharmaceutical Sciences Division, School of Pharmacy, University of Wisconsin-Madison, Madison, WI USA; 2 Cancer Pharmacology Lab, University of Wisconsin Carbone Cancer Center, Madison, WI USA; 3 Department of Oncology, McArdle Laboratory for Cancer Research, University of Wisconsin-Madison School of Medicine and Public Health, Madison, WI USA; 4 Department of Human Oncology, University of Wisconsin-Madison School of Medicine & Public Health, Madison, WI USA; 5 Present Address: Biology Discovery, Denali Therapeutics, 161 Oyster Point Boulevard, South San Francisco, CA 94080 USA; 6 Present Address: Sanders-Brown Center on Aging, College of Medicine, University of Kentucky, Lexington, KY USA; 7 Present Address: Department of Pharmaceutics, College of Pharmacy, University of Minnesota-Twin Cities, Minneapolis, MN USA

**Keywords:** Blood-brain barrier, Drug delivery, CNS cancer

## Abstract

Perillyl alcohol (POH) has been extensively studied for the treatment of peripheral and primary brain tumors. The intranasal route of administration has been preferred for dosing POH in early-stage clinical trials associated with promising outcomes in primary brain cancer. However, it is unclear how intranasal POH targets brain tumors in these patients. Multiple studies indicate that intranasally applied large molecules may enter the brain and cerebrospinal fluid (CSF) through direct olfactory and trigeminal nerve-associated pathways originating in the nasal mucosa that bypass the blood–brain barrier. It is unknown whether POH, a small molecule subject to extensive nasal metabolism and systemic absorption, may also undergo direct transport to brain or CSF from the nasal mucosa. Here, we compared CSF and plasma concentrations of POH and its metabolite, perillic acid (PA), following intranasal or intravascular POH application. Samples were collected over 70 min and assayed by high-performance liquid chromatography. Intranasal administration resulted in tenfold higher CSF-to-plasma ratios for POH and tenfold higher CSF levels for PA compared to equal dose intravascular administration. Our preclinical results demonstrate POH undergoes direct transport from the nasal mucosa to the CSF, a finding with potential significance for its efficacy as an intranasal chemotherapeutic for brain cancer.

## Introduction

The intranasal (IN) route of administration has long been appreciated as an option for local and systemic delivery for certain small molecules, peptides, and protein drugs^1,2^. In particular, the nasal route possesses distinct advantages when local effects are needed (e.g. as with decongestants, antibiotics, and mucolytics) or when non-invasive access to the systemic circulation is required for fast drug onset and/or to avoid extensive hepatic first-pass elimination (e.g. as with the application of the opioid antagonist naloxone following opioid overdose). Multiple clinical studies show that intranasally applied small molecules (e.g. zolmitriptan, sumatriptan, butorphanol tartrate, fentanyl, nicotine, and estradiol) and low molecular weight peptide drugs (e.g. calcitonin, desmopressin, buserelin, oxytocin), can achieve improved systemic exposure and therapeutically relevant concentrations in peripheral tissues^3^. Improved systemic exposure for these molecules is likely due to greater paracellular permeability across the nasal epithelia and more efficient absorption into the bloodstream through the extensive nasal vasculature present in the underlying lamina propria^1,4^. In contrast, intranasal delivery results in improved central exposure to the brain or CSF for only a subset of small molecules^1,5–12^. Mechanistic studies indicate that intranasally applied large molecules can directly access the central nervous system (CNS) through olfactory or trigeminal nerve-associated pathways that originate within the nasal mucosa^13–17^. Collectively, these findings have increased clinical interest in using the intranasal route for enhanced central delivery of small molecules. One such small molecule is perillyl alcohol (POH), a 152 Da plant-derived monocyclic terpene and chemotherapeutic agent. In vitro studies have demonstrated that both POH and its most stable metabolite, perillic acid (PA), may inhibit inflammation and cell proliferation by inducing apoptosis and inhibiting protein prenylation, a post-translational modification that ensures membrane-bound proteins are transported from the cytoplasm to the cell membrane to exert their function^18^. Importantly, POH is being actively investigated in clinical trials for the treatment of recurrent forms of primary brain cancers, particularly low-grade glioma (NCT02704858)^19–22^. POH was initially studied as an orally applied chemotherapeutic that showed efficacy in hepatic and mammary tumor-bearing rodents^23,24^. However, toxicity findings in multiple phase I clinical trials indicated that orally applied POH was associated with dose-limiting gastrointestinal toxicity^25–28^. Intranasally applied POH, alternatively, minimized these toxicity issues and sensitized the lesions to standard chemotherapeutic treatments such as surgical excision, radiotherapy, or chemotherapy^19–21^. Moreover, chronic intranasal application of POH blocks cell proliferation and migration pathways in cell cultures and significantly reduces tumor volumes in brain tumor xenograft-bearing mouse models^29–31^. More recent preclinical studies have also suggested that intranasally and intra-arterially applied POH may reversibly open the blood–brain barrier to some degree, resulting in improved CNS exposure of chemotherapeutics such as bortezomib, temozolomide, methotrexate, anti-PD-1, and CAR-T in xenograft-derived tumor-bearing rodents^32,33^. In clinical trials, primary brain cancer patients receiving intranasal POH every week for over 16 months have significantly reduced tumor volumes compared to tumor volumes at baseline (i.e. scans performed at week 0) ^19–21^.

Despite this surge in interest in utilizing POH as an intranasally delivered chemotherapeutic for brain cancers, the question of whether this dosing route is important for its clinical use is limited by several unknowns. First, POH concentrations in brain and/or CSF following intranasal administration have not been reported in the literature. Second, it is yet unclear how intranasally applied POH achieves therapeutically relevant concentrations within the CNS. Third, it is not known whether intranasally applied POH gains access to the brain and/or CSF from the systemic circulation after crossing the blood–brain and blood-CSF interfaces or, alternatively, if POH may reach the brain and/or CSF through direct olfactory and trigeminal nerve associated pathways originating in the nasal mucosa^2,13,14,34^. Here, we compared pharmacokinetic profiles for POH and its major metabolite following intranasal and intravascular POH administration in female, wild-type Sprague–Dawley rats. Our study demonstrates that the intranasal route is associated with a unique CSF exposure advantage compared to intravascular dosing for POH, a finding of importance for the clinical application of the intranasal route with POH, POH-drug conjugates and chemically modified POH analogs in the treatment of primary brain cancer patients.

## Results

**Figure 1:**
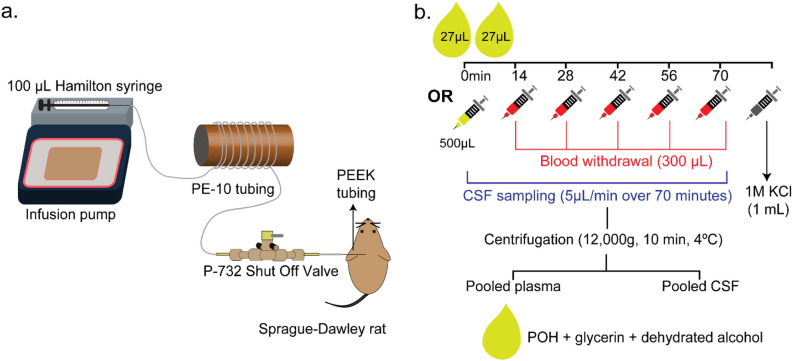
Experimental setup. **(a)** Surgical setup for intracisternal withdrawal of cerebrospinal fluid. **(b)** Dosing paradigm for intranasal and intravascular perillyl alcohol (POH) administration experiments. Rats were euthanized by intracardiac injection of 1 M potassium chloride (KCl) after the final blood and cerebrospinal fluid (CSF) collection steps.

**Figure 2:**
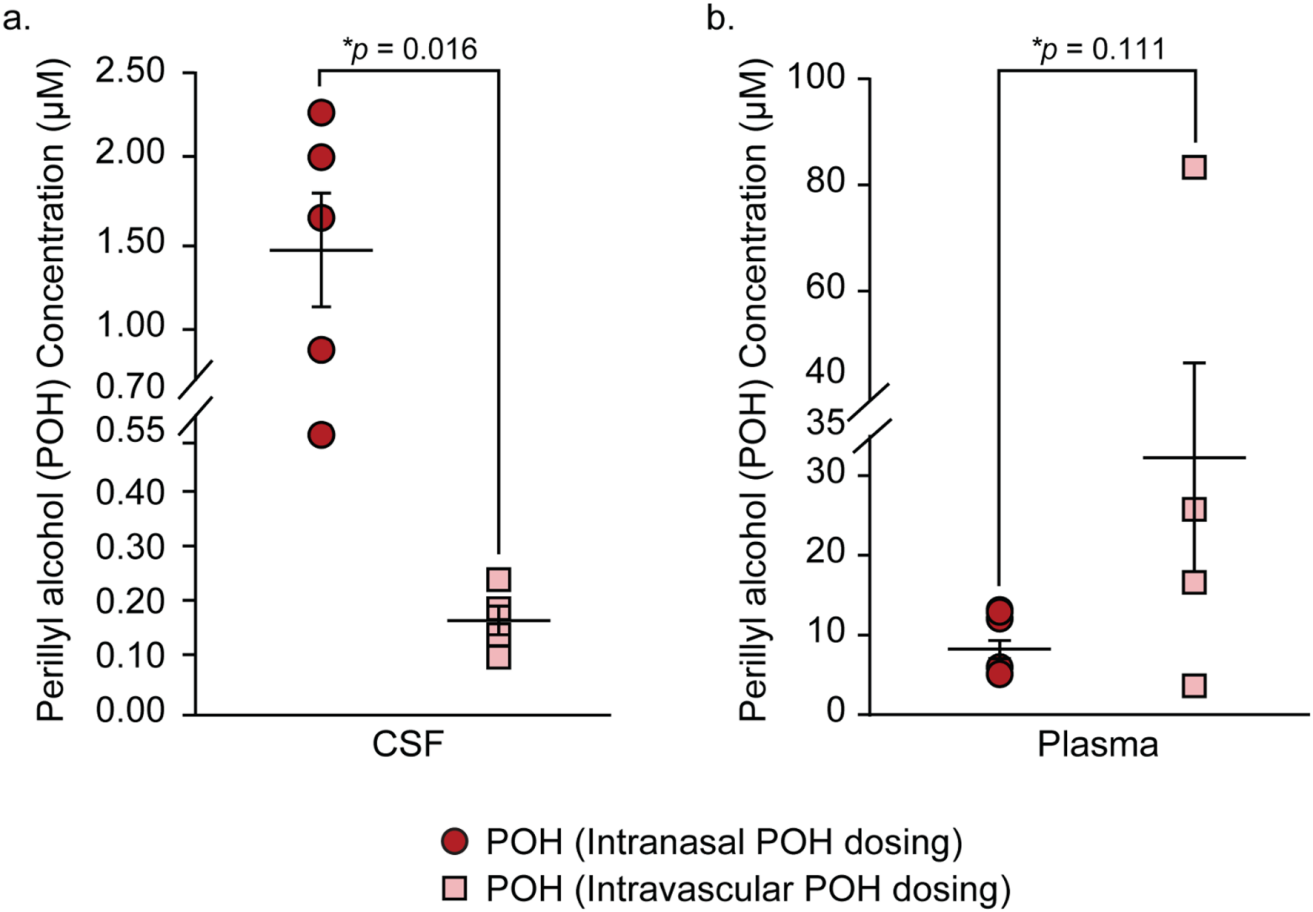
Average POH concentration in the CSF and plasma following equal-dose intranasal and intravascular POH administration. **(a)** Concentration of POH in the rat CSF following intranasal (red circles) and intravascular (pink squares) POH administration. **(b)** Concentration of POH in the rat plasma following intranasal (red circles) and intravascular (pink squares) POH administration. *p values calculated by Mann–Whitney rank sum test.

**Figure 3:**
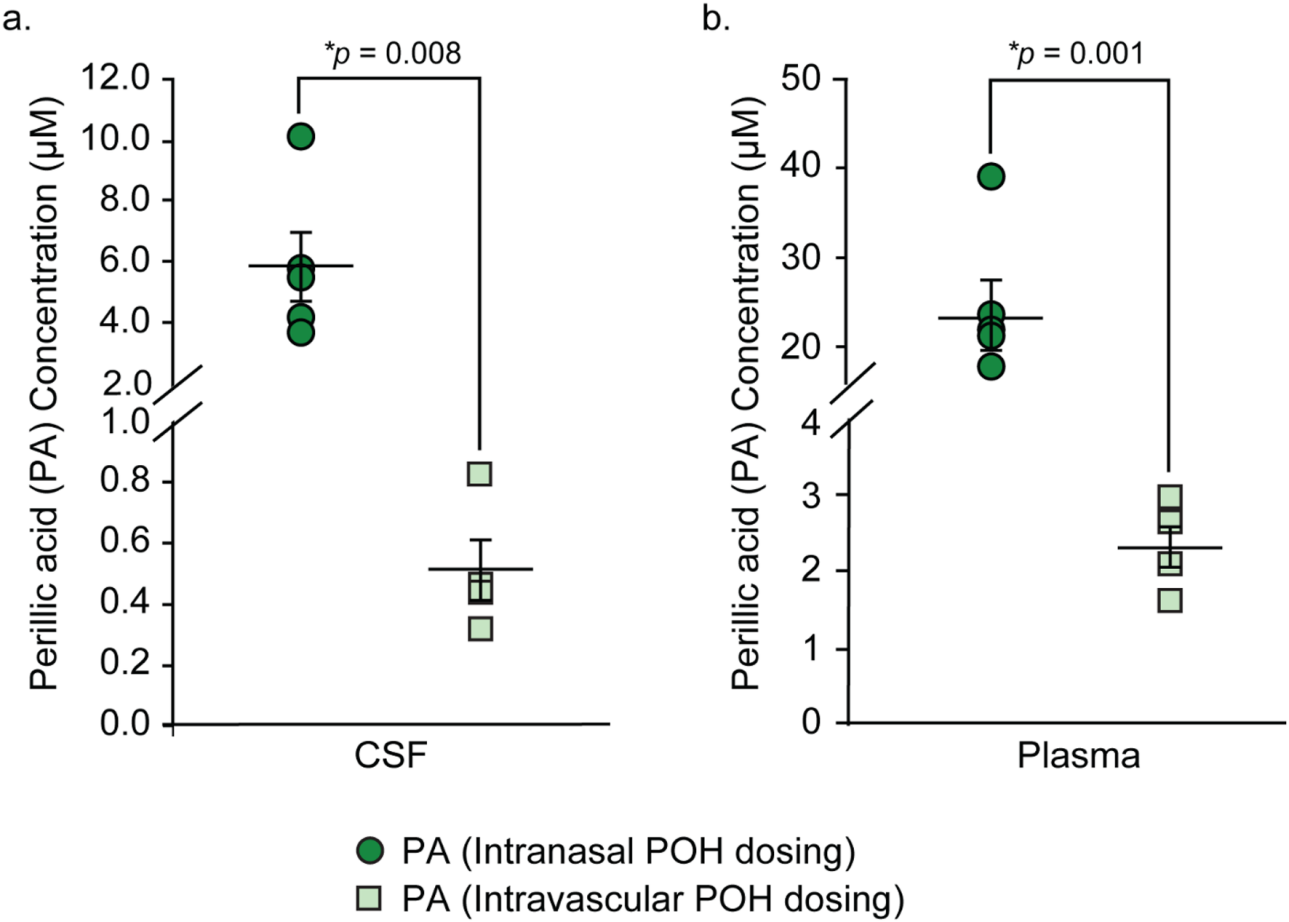
Average PA concentration in the CSF and plasma following equal-dose intranasal and intravascular POH administration. **(a)** Concentration of PA in the rat CSF following intranasal (dark green circles) and intravascular (light green squares) POH administration. **(b)** Concentration of PA in the rat plasma following intranasal (dark green circles) and intravascular (light green squares) POH administration. *p values calculated by Mann–Whitney rank sum test **(a)** or Student’s t-test **(b)**.

The experimental setup and study design are illustrated in Fig. 1. Adult female Sprague–Dawley rats with an average weight of 210 g received an equal dose of POH via intranasal (*n* = 5) or intravascular (n = 4) administration. The fixed dose administered to animals in both groups (4.745 mg) was chosen to represent: (i) the lowest POH dose described in previous preclinical studies and (ii) the allometrically-scaled dose corresponding to the intranasal POH formulation from an ongoing clinical trial examining its safety and efficacy in glioma patients (NCT02704858)^29,35–38^ (see “Methods” for further description). The intranasal POH formulation was directed to a region near the olfactory mucosa to increase the likelihood of CNS delivery since the olfactory mucosa has relatively lower vascular permeability and mucociliary clearance compared to the nasal respiratory mucosa^4,13^. CSF and plasma samples were processed to precipitate residual proteins (e.g. albumin) and to allow for total (bound and unbound) POH in each compartment to be analyzed. CSF concentration of POH was below the lower limit of detection (LLOD) for the assay in 1 out of 5 animals in the intravascular POH cohort; these values were therefore excluded from data analysis. POH and PA concentrations in the plasma and CSF were above LLOD for all other cases (Figs. 2, 3). Table 1 indicates AUC_70_ values for this study, i.e. total analyte concentrations over 70 min of POH administration. Tables 2 and 3 summarize mean concentrations and CSF-to-plasma ratios. These results are also discussed in the following sections.

**Table 1:** Total analyte exposure or AUC_70_.

	Intranasal POH dosing (µM min)	Intravascular POH dosing (µM min)
POH (plasma)	529.9 ± 123.9	2317.7 ± 1104.6
POH (CSF)	104.3 ± 23.8	11.2 ± 2.1
PA (plasma)	1674.4 ± 276.5	165.9 ± 18.9
PA (CSF)	411.6 ± 79.8	35.7 ± 7.0

Total drug exposure values for POH and PA in the rat plasma and CSF over 70 min of experimental duration. Values are depicted as mean ± standard error of mean (SEM).

*AUC*_*70*_ area under the concentration–time curve over the experimental timeframe (70 min).

**Table 2:** Mean analyte concentrations.

	Intranasal POH dosing (µM)	Intravascular POH dosing (µM)
POH (plasma)	7.57 ± 1.77	33.11 ± 15.78
POH (CSF)	1.49 ± 0.34	0.16 ± 0.03
PA (plasma)	23.92 ± 3.95	2.37 ± 0.27
PA (CSF)	5.88 ± 1.14	0.51 ± 0.10

Mean analyte concentrations for perillyl alcohol (POH) and perillic acid (PA) in the rat plasma and cerebrospinal fluid (CSF). Values are depicted as mean ± standard error of mean (SEM).

**Table 3:** CSF/plasma ratios.

	Intranasal POH dosing	Intravascular POH dosing
POH	0.20 ± 0.01	0.02 ± 0.01
PA	0.26 ± 0.02	0.21 ± 0.02

CSF/plasma ratios for perillyl alcohol (POH) and perillic acid (PA) following intranasal and intravascular POH administration. Values are depicted as mean ± standard error of mean (SEM).

### Plasma and CSF exposure for perillyl alcohol and perillic acid following intravascular application

**Figure 4:**
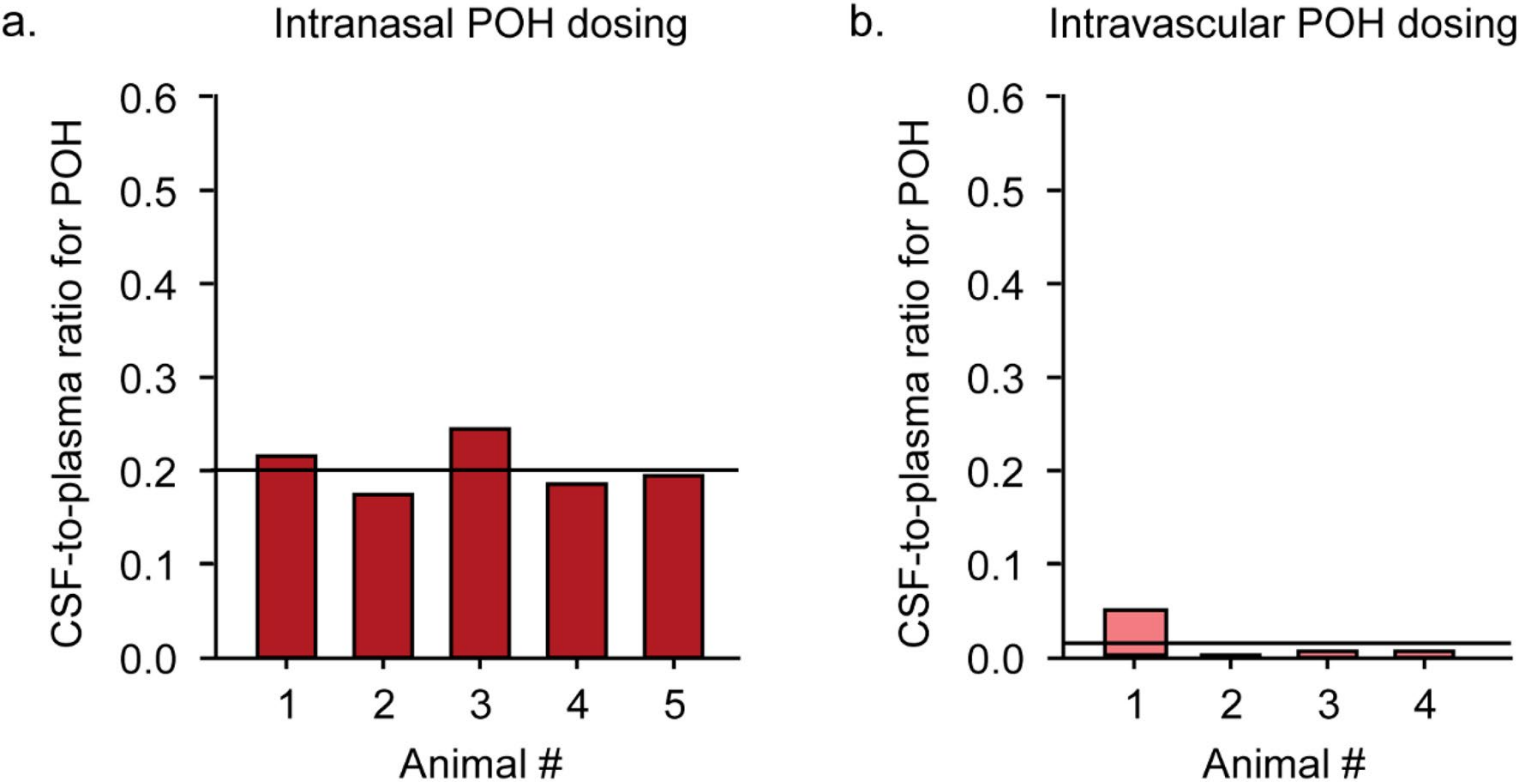
CSF-to-plasma ratio for POH following intranasal **(a)** or intravascular **(b)** POH administration. Values for intranasal and intravascular POH administration are shown in red bars **(a)** and pink bars **(b)** respectively. Solid lines indicate the mean values for each group.

Intravascular application of POH to female Sprague–Dawley rats (n = 4) resulted in a mean POH plasma concentration of 33.11 ± 15.78 µM (Fig. 2b; Table 2) and a mean POH plasma AUC_70_ of 2317.7 ± 1104.6 µM.min (Table 1). CSF exposure for POH in these animals was approximately 200-fold-lower than plasma POH exposure, with a mean POH CSF concentration of 0.16 ± 0.03 µM (Fig. 2a; Table 2) and a mean POH CSF AUC_70_ value of 11.2 ± 2.1 µM.min (Table 1). The mean CSF-to-plasma ratio for POH was 0.02 ± 0.01 following intravascular application (Fig. 4b; Table 2).

**Figure 5:**
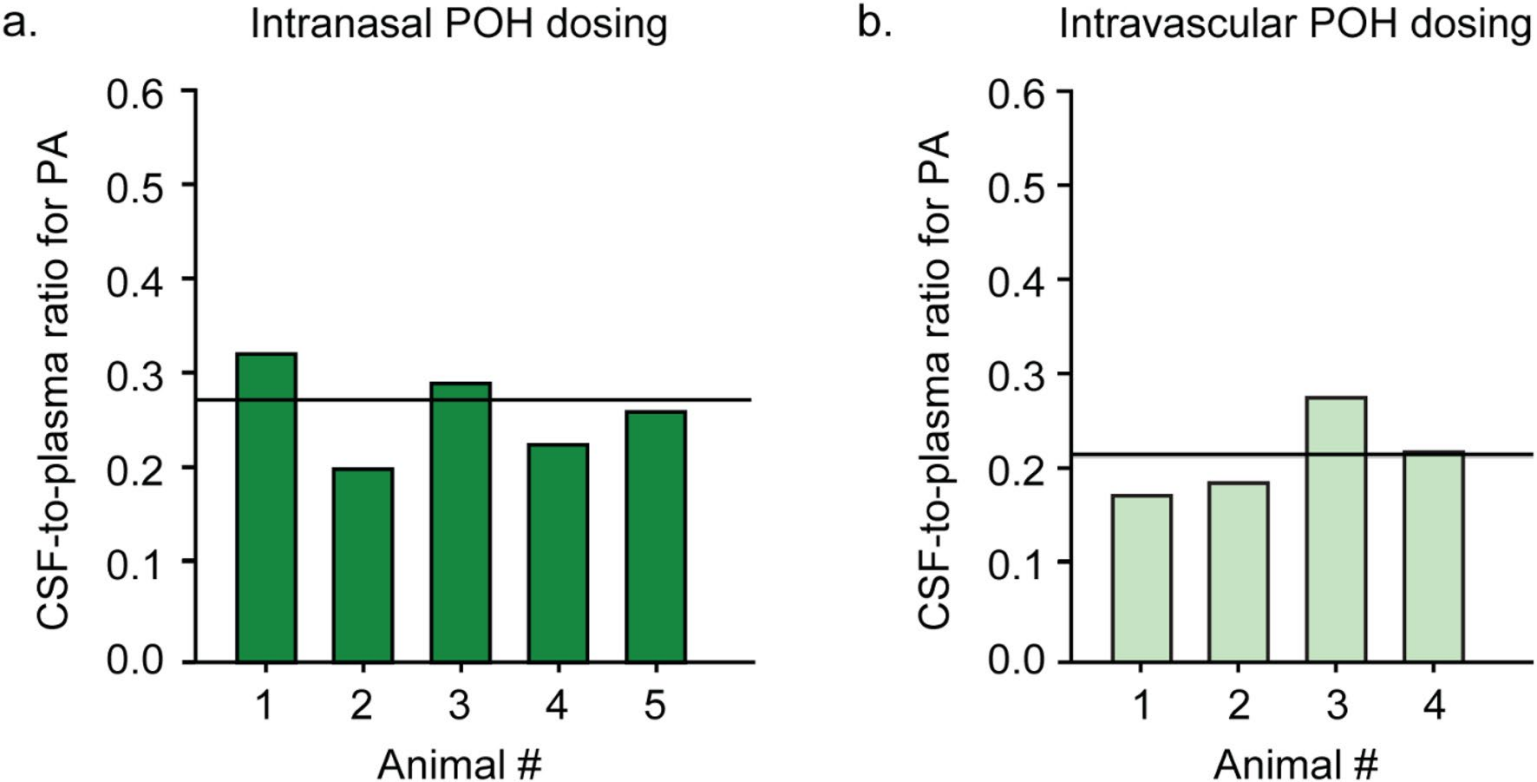
CSF-to-plasma ratio for PA following intranasal **(a)** or intravascular **(b)** POH administration. Values for intranasal and intravascular POH administration are shown in dark green bars **(a)** and light green bars **(b)** respectively. Solid lines indicate the mean values for each group.

Apart from POH, perillic acid (PA), the major POH metabolite, was also assessed in both CSF and plasma taken from the same animals. Intravascularly applied POH resulted in a mean PA plasma concentration of 2.37 ± 0.27 µM (Fig. 3b; Table 2) and mean PA plasma _AUC70_ value of 165.9 ± 18.9 µM.min (Table 1). CSF PA exposure was approximately fourfold lower than plasma PA exposure, with a mean PA CSF concentration of 0.51 ± 0.10 µM (Fig. 3a; Table 2) and a mean PA CSF AUC_70_ value of 35.7 ± 7.0 µM.min (Table 1). Mean CSF-to-plasma ratio for PA was 0.21 ± 0.02 in this group (Fig. 5b; Table 3).

### Enhanced CSF exposure for perillyl alcohol and metabolite following intranasal application

Intranasal application of POH to female Sprague–Dawley rats (n = 5) resulted in a mean POH plasma concentration of 7.57 ± 1.77 µM (Fig. 2b; Table 2) and a mean POH plasma _AUC70_ of 529.9 ± 123.9 µM.min (Table 1). CSF exposure in these animals was remarkably only about fivefold lower than plasma, with a mean POH CSF concentration of 1.49 ± 0.34 µM (Fig. 2a; Table 2) and a mean POH CSF AUC_70_ value of 104.3 ± 23.8 µM min (Table 1). These values resulted in a CSF-to-plasma ratio of 0.20 ± 0.01 (Fig. 4a; Table 2). In other words, intranasal administration of POH resulted in a tenfold higher CSF-to-plasma ratio compared to intravascularly applied POH, consistent with a significant degree of direct targeting of POH to the CSF from the nasal passage.

Animals that received POH intranasally also had tenfold higher PA metabolite concentrations in the CSF and plasma compared to animals that received intravascular POH. Specifically, intranasal application of POH resulted in a mean PA plasma concentration of 23.92 ± 0.24 µM (Fig. 3b; Table 2) and a mean PA plasma AUC_70_ value of 1674.4 ± 276.5 µM min (Table 1). These values were approximately threefold higher than mean POH plasma values in the same animals, consistent with significant nasal metabolism and subsequent PA systemic absorption from the nasal mucosa. CSF PA exposure in animals that received POH intranasally was approximately fourfold lower than plasma PA exposure, with a mean PA CSF concentration of 5.88 ± 1.14 µM (Fig. 3a; Table 2) and a mean PA CSF AUC_70_ value of 411.6 ± 79.8 µM min (Table 1). Collectively, these values suggest that intranasally applied POH is rapidly metabolized to PA, resulting in elevated PA levels in both plasma and CSF within 70 min of intranasal application. The mean CSF-to-plasma ratio for PA was 0.26 ± 0.02 in these animals (Fig. 5a; Table 3), which was similar to the mean CSF-to-plasma ratio for PA in the intravascular group (Fig. 5b; Table 3).

## Discussion

This study has yielded several significant new findings: (i) intranasally applied POH results in a tenfold higher CSF-to-plasma ratio for POH compared to intravascular controls, (ii) intranasally applied POH results in tenfold higher PA concentrations in the CSF and plasma compared to intravascular delivery, and (iii) intranasal and intravascular application of POH resulted in similar CSF-to-plasma ratios for PA. Taken together, our results suggest intranasal POH application is more suited to obtain greater CSF exposure for POH and PA than intravascular POH application. Most importantly, our results provide new evidence supporting significant direct transport from the nasal mucosa to the CSF for POH, suggesting a strong advantage for the intranasal route in the central delivery of POH.

Our group has previously described how intranasally applied macromolecules access the CNS via potential pathways originating in the nasal mucosa^2,4,13,16,39,40^. Briefly, intranasally applied molecules may transit through the olfactory and/or respiratory nasal epithelia via intracellular or extracellular pathways to reach the underlying connective tissue^2,4,13,16,39,40^. Once within the lamina propria, molecules may experience several different fates: (i) systemic absorption into the nasal vasculature to enter the systemic circulation, (ii) lymphatic absorption into draining nasal lymphatic vessels and downstream cervical lymph nodes, and/or (iii) direct transport along pathways associated with the olfactory and trigeminal nerves (perivascular or perineural) to enter the CSF and brain. More detailed mechanistic studies by our group have shown that macromolecules enter the CNS by direct transport pathways at the level of the olfactory bulbs (via olfactory pathways) or the brainstem (via trigeminal pathways) ^2,4,13,16,39,40^. Subsequent widespread distribution from these regions may then occur within the perivascular spaces around cerebral blood vessels via convective transport or dispersion potentially down to the capillary level^14,39,41^.

Based on its molecular weight, POH (152 Da) belongs to a subset of non-polar small molecules that in theory may passively diffuse through the phospholipid bilayers of intact brain capillary endothelial cells to enter the CNS^42^. POH also has a high binding affinity for albumin (*K*_d_ = 0.19 M) that results in a relatively low unbound drug fraction within albumin-rich fluid compartments such as plasma or CSF (unbound fraction, fu ~ 0.2—0.4)^43^. Albumin-POH interactions may therefore affect passive diffusion of POH across the blood–brain and blood-CSF barriers. Interestingly, the distribution of POH and its metabolites between the blood and CSF compartments has not been carefully examined.

In the present study, intranasally applied POH resulted in approximately fivefold lower POH concentrations in the CSF than that in plasma sampled from the same animals, indicating that POH is more likely to enter the systemic circulation following intranasal delivery. However, intravascularly applied POH at the same dose resulted in ~ 200-fold lower POH concentrations in the CSF than that in the plasma, indicating that a markedly higher relative POH CSF exposure was obtained with the intranasal route. These findings suggest that the intranasal route provides direct and elevated central exposure to POH.

Wang et al. recently reported a blood–brain barrier permeability increase following intracarotid POH application at doses down to ~ 800 mg/kg in 3-week-old C57BL/6 tumor-bearing mice, resulting in elevated CNS exposure for bloodborne molecules^32^. In contrast, an increase in blood–brain barrier permeability was not observed following POH application to intravascular sites other than to the carotid artery^32^. In our study, we administered a ~ 34-fold smaller dose (23 mg/kg) via the abdominal aorta and observed that POH concentrations in the CSF were 200-fold lower than POH concentrations in the plasma. Based on these findings, we do not believe that POH administration at the dose used in our study resulted in any significant effect on blood–brain barrier permeability.

We further observed that measured PA concentrations were greater than POH concentrations in all physiological compartments assessed (CSF, plasma) following intranasal dosing. This pattern of higher PA than POH levels was also observed for brain lysate following intranasal dosing of POH in a recent study^33^. Collectively, these findings from our study and Wang et al. suggest significant and rapid metabolism of POH to PA occurs following intranasal POH administration. Significant POH metabolism following intranasal application may be due to rapid POH-to-PA conversion at the nasal mucosa as well as along olfactory and trigeminal nerve-associated pathways into the CNS. POH is sequentially metabolized to perillaldehyde by alcohol dehydrogenases and then to perillic acid by aldehyde dehydrogenases. Prior studies have established that POH can also be sequentially altered to PA by cytochrome P450 and aldehyde dehydrogenases^44–46^. Interestingly, multiple reports indicate that these transmembrane enzymes are highly expressed in the olfactory mucosa at levels that exceed those within the respiratory mucosa^47–51^. Furthermore, significant alcohol dehydrogenase immunoreactivity appears to be associated with the perivascular compartment of systemic blood vessels, particularly smooth muscle cells of the tunica media^52^. High mRNA expression levels of alcohol and aldehyde dehydrogenases have also been detected at brain-CSF interfaces such as the leptomeninges, choroid plexus, and ependyma, as well as within the brain parenchyma^53–55^. The prominent presence of these biotransformative enzymes at the site of nasal administration, particularly the nasal region associated with brain delivery pathways (e.g. the olfactory epithelium), as well as at downstream sites that have been implicated in direct nose-to-brain biodistribution (e.g. the perivascular compartment of leptomeningeal blood vessels and brain tissue itself) is consistent with the significant POH-to-PA conversion we and others have observed following intranasal dosing.

Despite marked differences in POH CSF exposure, we observed that both intranasal and intravascular POH application resulted in similar CSF-to-plasma ratios for PA. One explanation for this result would be if PA were a substrate for specific transporters at the blood-CSF and blood–brain interfaces. This might lead to some degree of PA equilibration between blood and CSF irrespective of the delivery route and differences in metabolism. Indeed, immunohistochemical staining in the mouse has shown that brain microvessels, choroid plexus, and the glia limitans are highly immunoreactive for monocarboxylate transporters (MCT-1 and MCT-2)^56,57^, for which PA may well be a substrate. Further work will be needed to validate this hypothesis. Clarification of the regional biodistribution of both POH and PA in the brain following intranasal and intravascular dosing may reveal important sites for POH metabolism and PA transport; however, this was beyond the scope of the present study.

Our study has some limitations. Effects on POH biodistribution related to gender, species-differences, and pathology (e.g. tumor) will require further study. CSF and plasma samples were pooled over 70 min to obtain sufficiently large injection volumes for our HPLC setup. Investigating POH concentrations with more sophisticated detection systems may be able to yield more detailed kinetic information not provided in the present study. We also observed relatively high variability in measured POH plasma concentrations after intravascular dosing; the reasons for this variability were not entirely clear. Finally, the use of tracheotomized rats was necessary to ensure more consistent tolerance for the highly viscous POH formulation and to better allow for targeting to the olfactory mucosal area with tracer dosing. While unlikely, this experimental preparation may have had some effect on the resulting transport and central biodistribution observed following intranasal POH. Nevertheless, our results provide among the first evidence supporting intranasal enhancement of central POH exposure by a direct path that bypasses the blood–brain barrier. Our findings support further use of the intranasal route for the delivery of POH, POH-derived analogs, and possibly other similar chemotherapeutic agents to the CSF and brain for neuro-oncology applications.

## Materials and methods

### Standards and quality controls

S-(−)-Perillyl alcohol (POH; Catalog #218391) was obtained from Millipore-Sigma (St. Louis, MO, USA). Perillic acid (PA) was synthesized by Dr. Gould and colleagues based on methods described previously^57^. Acetonitrile (Catalog #A998-4) and methanol (Catalog #A452-4) were obtained from Fisher Scientific (Hampton, NH, USA). Commercially available Sprague–Dawley rat plasma (Catalog #50642312; Fisher Scientific) and artificial cerebrospinal fluid (ACSF; 124 mM NaCl, 3 mM KCl, 26 mM NaHCO_3_, 1.25 mM NaH_2_PO_4_, 1.3 mM MgCl_2_, 1.5 mM CaCl_2_ and 10 mM D-glucose equilibrated with 95% O2/ 5% CO2; 300 ± 5 mOsm/kg osmolality determined using a freezing-point osmometer. Model 3250 Osmometer (Advanced Instruments, Norwood, MA, USA) were used for assay calibration and validation. POH and PA were dissolved in methanol at a stock concentration of 10 µg/mL and 1000 µg/mL respectively and stored as one-time-use aliquots at -20 °C. POH standards were prepared in plasma and/or artificial CSF by serially diluting stock solutions (1.0, 0.75, 0.5, 0.4, 0.3, 0.2 and 0.1 µg/mL final concentrations). PA standards were prepared in a similar manner as mentioned above at 5.0, 4.0, 3.0, 2.0, 1.0, 0.75, 0.5, and 0.25 µg/mL final concentrations. Quality controls were prepared at 0.8, 0.45, and 0.25 µg/mL final concentrations for POH and 4.5, 2.5, and 0.65 µg/mL final concentrations for PA respectively.

### Sample preparation for chromatography

HPLC assay design was based on a previously described pharmacokinetic study by Hua and colleagues^35^ with slight modifications. Briefly, a 50 µL volume of sample/standard/quality control was supplemented with 100 µL acetonitrile and vortexed vigorously for 3 min (POH) or 1 min (PA). Samples were then centrifuged (14,000 *g*, 4 °C, 10 min for POH; 14,000 *g*, room temperature, 5 min for PA). Following centrifugation, 20 µL of supernatant was injected into the HPLC system. For PA analysis, 120 µL of supernatant was carefully transferred to a clean tube and dehydrated under a stream of nitrogen. Pellets were then resuspended in 100 µL diluted mobile phase (50% mobile phase, 50% acetonitrile, 10 mM NaHCO_3_), and 80 µL of this resuspension was injected into the HPLC system.

### Chromatography setup

The chromatography setup comprised of Shimadzu Prominence HPLC system modules, a degasser, an auto-sampler, a UV detector (wavelengths set to 210 nm and 217 nm for POH and PA detection respectively), and a column oven (Shimadzu Scientific Instruments Inc., Columbia, MD, USA). The separation was performed using a Zorbax Eclipse XDB-C18 column (4.6 × 150 mm, 5 µm; Agilent Technologies, Santa Clara, CA, USA) and a Zorbax C18 guard (8 × 4 mm, 5 µm; Agilent Technologies, Santa Clara, CA, USA). Mobile phase composition for both analytes was isocratic, comprising of water–acetonitrile mixture (60:40, v/v, pre-mixed, 1.0 mL/min) for POH analysis and 0.05 M ammonium acetate (pH 5)–acetonitrile mixture (64:36, v/v, pre-mixed, 2.0 mL/min) for PA analysis. Both phases were passed through 0.45 µm filters (Millipore-Sigma, St. Louis, MO, USA) before use.

### POH formulation

POH formulation used for this study was adapted from the nasal formulation recipe used in an ongoing clinical trial that examines the safety and efficacy of perillyl alcohol in recurrent grade IV glioma patients (NCT02704858). Briefly, intranasal POH formulation consisted of 10% v/v (9.4% w/w) POH, 55% v/v glycerin USP and 35% v/v dehydrated alcohol. Dose for in vivo experiments was determined by allometric scaling based on body weight^37^ to determine doses for rats based on the smallest daily dose received by a human subject in the clinical trial i.e. 4.745 mg (i.e. 23.75 mg/kg). Formulations were refrigerated as one-time-use aliquots (54 µL per aliquot) at 4 °C. The volume (54 µL) was considered safe for intranasal administration based on experiences with similar volumes for intranasal study paradigms in rats in our lab^4,14,39^.

### Intracisternal sampling of cerebrospinal fluid (CSF)

A dedicated CSF withdrawal system was used for POH experiments (Fig. 1). This system comprised of a 20 cm long polyethylene tubing (PE-10; inner diameter: 0.61 mm, outer diameter: 0.28 mm; Plastics One Inc., Roanoke, VA, USA); a 100 µL Hamilton syringe (inner diameter: 1.475 mm; Harvard Apparatus, Holliston, MA, USA), and a two-port shut-off valve assembly (PEEK, inner diameter: 0.5 mm; Idex Health and Science, Oak Harbor, WA, USA). Tubing was firmly secured at both ends of the valve assembly using NanoTight sleeves (FEP; inner diameter: 0.69 mm; Idex Health and Science, Oak Harbor, WA, USA); these sleeves flanked on either side of valve fittings. The valve fitting side that faced the cisternal space was connected to a different set of tubing assembly i.e. 1 cm PE-10 tubing sealed to a 1.27 mm long polyether ether ketone tubing (PEEK; 33 GA; Plastics One Inc., Roanoke, VA, USA). PE-10/PEEK tubing connections were sealed using molten wax and cyanoacrylate on the day of the experiment.

### Ethics approval

All animal experiments were conducted at the University of Wisconsin-Madison as per the NIH Guide for the Care and Use of Laboratory Animals (8th edition, 2011), the ARRIVE guidelines, and following the approval of the Institutional Animal Care and Use Committee regulations at the University of Wisconsin-Madison. Animals were housed under 12 h light/12 h dark cycles and climate-controlled conditions with ad libitum access to food and water. All experiments were terminal, and efforts were made to ensure minimal pain and distress during anesthesia and cannulations.

### Animal preparation

Adult female Sprague Dawley rats (mean weight 210 g; 4-month-old; Envigo/Harlan, Indianapolis, IN) were anesthetized under urethane (1.5 g/kg, i.p.) with boosters to effect. Atropine sulfate (0.1 mg/kg, i.m.) was given every 2 h to avoid fluid accumulation in the lungs. Animals were maintained at physiological body temperature using a generic homeothermic monitoring system throughout the experiment.

### Tracheotomy (prior to intranasal POH administration)

Preliminary experiments indicated that glycerin was increasing the viscosity of POH formulation (ethylene glycol: 0.0162 N s/m^2^, glycerin: 0.95 N s/m^2^) that resulted in breathing issues and low survival rate of animals at the experimental endpoint (70 min). Hence, animals subjected to intranasal POH administration underwent a brief tracheotomy procedure before cisternal puncture. Briefly, an incision was made parallel to rings of hyaline cartilage in the anterior tracheal region; a short piece (5 cm) of PE-205 tubing (outer diameter: 2 mm) was then inserted into this opening. Tubing was secured by 2–3 surgical knots with a general-purpose thread, followed by sealing the skin with cyanoacrylate. In our experience, tracheotomy prior to intranasal administration increased the survival rate and time for rats. Tracheotomy was not performed for intravascular POH experiments.

### Intracisternal cannulation

Intracisternal cannulation was the first procedure conducted on animals undergoing intravascular POH dosing. Cannulation was performed after the tracheotomy procedure for intranasal POH administration experiments. Cisternal cannulation procedure was adapted from a recently reported setup for intrathecal delivery in rats at our laboratory^37^. Briefly, lidocaine hydrochloride (2%; 0.5 mL, s.c.) was applied as a local anesthetic at the scalp before positioning the animals in a stereotaxic frame. A 5 cm midline incision was then made from the base of the neck 2 cm anterior to the bregma. The skin was laterally secured with hemostats and muscle layers were sequentially retracted using vannas scissors and cotton tip applicators. Once the atlanto-occipital layer was exposed, gentle incisions were made by alternating between a goniotomy knife and forceps to avoid dural puncture. When the cisterna magna was visible beneath the intact dura, a small dural hole was made using a dental needle at a 60° angle from the vertical. PEEK tubing was then inserted with the help of a microelectrode holder and stereotaxic frame’s manipulator arm. Excess CSF flowing out from the hole was absorbed using cotton tip applicators and tubing was sealed to the cranial opening with cyanoacrylate; sealed setup was then dried until cured.

### Abdominal aortic cannulation

Intracisternal cannulation preceded abdominal aortic cannulation for both treatment groups. Briefly, animals were carefully flipped over to the ventral side without displacing the tubing from its position. An incision was made below the xiphoid process along the abdominal midline. Skin and visceral organs were sequentially retracted to obtain a clear view of the aorta. Aorta was separated from the vena cava by cotton tip applicators and curved sharp forceps. The fascia between the two abdominal vessels was broken the opening was widened. Next, a 6-inch long piece of general-purpose thread was tied to the bottom of this opening to cut off blood supply to the lower body. Another piece of thread (~ 12 in) was loosely held at the top of this opening to secure a 20 GA, 1.25-inch cannula attached to a 3-way stop cock.

### Intranasal administration paradigm

Aliquots of 54 µL of POH formulation were intranasally applied using 200 µL gel loading pipette tips. Briefly, 17 mm of a gel loading pipette tip was inserted into one naris (45° from the midline, 45° from the horizontal plane). Once the tip was 5 mm inside the nasal cavity, it was aligned parallel to the nasal septum bone and inserted 12 mm further into the cavity. The formulation was pipetted close to the olfactory nasal mucosa, avoiding any tissue damage. Within the first 2 min, 2 bolus drops of intranasal POH formulation (27 µL each) were administered to each naris of the animal.

### Intravascular administration paradigm

For intravascular application, the POH formulation was diluted to 0.5 mL (final concentration 0.92% w/v) prior to administration. Briefly, 54 µL of POH formation was diluted with 445 µL of physiological saline (150 mM NaCl, 0.9%, pH 7.4) and injected into the abdominal aorta using a 3-way stop-cork. Another round of 500 µL saline was injected to flush the entire formulation from the cannula into the artery.

### Blood and CSF withdrawal

CSF was withdrawn at 0–12 min, 14–26 min, 28–40 min, 40–54 min, and 56–70 min at 5 µL/min. After each withdrawal, CSF samples were ejected from the tubing into clean microcentrifuge tubes over a 2-min interval. Tubes were pre-cooled on ice. Blood sampling (300 µL) was performed every 14 min. Blood was collected in 1.5 mL microcentrifuge tubes containing 3 µL sodium heparin (anticoagulant; 1000 USP units). Tubes were centrifuged (3000 rpm, 4 °C, 5 min) to separate red blood cells from the CSF and plasma at the end of the experiment. Samples were further spun (12,000 rpm, 4 °C, 5 min) to remove additional contamination. Samples were stored (− 80 °C) until ready for analysis. Before HPLC analysis, samples from each animal were thawed and pooled across all time points to run HPLC assays in triplicates.

### Euthanasia

Animals were euthanized by injecting 1 mL KCl (1 M) into the pericardium.

### Statistical analysis

For each HPLC analysis, LLOD were determined before sample analysis. Pure samples used for standard curves were dissolved in artificial CSF (aCSF) and commercially available rat plasma. POH concentrations in the CSF and plasma were each estimated by linear regression analysis. The CSF concentration of POH was below the LLOD for the assay in 1 out of 5 animals in the intravascular POH cohort; these values were therefore excluded from statistical analysis. POH and PA concentrations in the plasma and CSF were above LLOD for all other cases. Treatment groups were compared using the Student’s t-test when the two groups exhibited normal distribution and equal variance. In the absence of a normal distribution, groups were compared using the Mann–Whitney rank-sum test. Both analyses were performed using SigmaPlot (Systat Software, San Jose, CA) to identify statistical significance.

## References

[1] Costantino HR, Illum L, Brandt G, Johnson PH, Quay SC (2007). Intranasal delivery: Physicochemical and therapeutic aspects. Int. J. Pharm..

[2] Lochhead, J. J. & Thorne R. G. Intranasal drug delivery to the brain. in *Drug Delivery to the Brain* (ed. Hammarlund-Udanaes, M., de Lange, E. C. M. & Thorne, R. G.) 401–431. (Springer, 2013).

[3] Illum L (2012). Nasal drug delivery—Recent developments and future prospects. J. Control. Release..

[4] Kumar, N. N., Gautam, M., Lochhead, J. J., Wolak, D. J., Ithapu, V., Singh, V. & Thorne, R. G. Relative vascular permeability and vascularity across different regions of the rat nasal mucosa: Implications for nasal physiology and drug delivery. *Sci. Rep.* **6,** 31732, 10.1038/srep31732 (2016). 10.1038/srep31732 PMC4997340 27558973

[5] Sakane T, Akizuki M, Yoshida M, Yamashita S, Nadai T, Hashida M, Sezaki H (1991). Transport of cephalexin to the cerebrospinal fluid directly from the nasal cavity. J. Pharm. Pharmacol..

[6] Sakane T, Akizuki M, Yamashita S, Sezaki H, Nadai T (1994). Direct drug transport from the rat nasal cavity to the cerebrospinal fluid: The relation to the dissociation of the drug. J. Pharm. Pharmacol..

[7] Sakane T, Akizuki M, Taki Y, Yamashita S, Sezaki H, Nadai T (1995). Direct drug transport from the rat nasal cavity to the cerebrospinal fluid: The relation to the molecular weight of drugs. J. Pharm. Pharmacol..

[8] Kao HD, Traboulsi A, Itoh S, Dittert L, Hussain A (2000). Enhancement of the systemic and CNS specific delivery of L-dopa by the nasal administration of its water-soluble prodrugs. Pharm. Res..

[9] Chow, H. H., Anavy, N. & Villalobos, A. Direct nose-brain transport of benzoylecgonine following intranasal administration in rats. *J. Pharm. Sci.* **90 (11),** 1729–1735; https://doi.org/10.1002/jps.1122 (2001). 10.1002/jps.1122 11745730

[10] Al-Ghananeem AM, Traboulsi AA, Dittert LW, Hussain AA (2002). Targeted brain delivery of 17 beta-estradiol via nasally administered water-soluble prodrugs. AAPS PharmSciTech.

[11] Leonard AK, Sileno AP, MacEvilly C, Foerder CA, Quay SC, Costantino HR (2005). Development of a novel high-concentration galantamine formulation suitable for intranasal delivery. J. Pharm. Sci..

[12] Barakat NS, Omar SA, Ahmed AEA (2006). Carbamazepine uptake into rat brain following intra-olfactory transport. J. Pharm. Pharmacol..

[13] Lochhead, J. J. & Thorne, R. G. Intranasal delivery of biologics to the central nervous system. *Adv. Drug Deliv. Rev.* **64,** 614–628; 10.1016/j.addr.2011.11.002 (2012). 10.1016/j.addr.2011.11.002 22119441

[14] Lochhead, J. J., Wolak, D. J., Pizzo, M. E. & Thorne, R. G. Rapid transport within cerebral perivascular spaces underlies widespread tracer distribution in the brain after intranasal administration. *J. Cereb. Blood Flow Metab.* **35,** 371–381, 10.1038/jcbfm.2014.215 (2015). 10.1038/jcbfm.2014.215 PMC4348383 25492117

[15] Thorne, R. G., Emory, C. R.., Ala, T. A. & Frey, W. H. Quantitative analysis of the olfactory pathway for drug delivery to the brain. *Brain Res.* **692,** 278–282, 10.1016/0006-8993(95)00637-6 (1995). 10.1016/0006-8993(95)00637-6 8548316

[16] Thorne RG, Pronk G, Padmanabhan V, Frey WH (2004). Delivery of insulin-like growth factor-I to the rat brain and spinal cord along olfactory and trigeminal pathways following intranasal administration. Neuroscience.

[17] Thorne RG, Hanson LR, Ross TM, Tung D, Frey WH (2008). Delivery of interferon-b to the monkey nervous system following intranasal administration. Neuroscience.

[18] Mukhtar YM, Adu-Frimpong M, Xu X, Yu J (2018). Biosci. Rep..

[19] Da Fonseca CO, Silva JT, Lins IR, Simao M, Arnobio A, Futuro D, Quirico-Santos T (2009). Correlation of tumor topography and peritumoral edema of recurrent malignant gliomas with therapeutic response to intranasal administration of perillyl alcohol. Invest. New Drugs..

[20] Da Fonseca CO, Simao M, Lins IR, Caetano RO, Futuro D, Quirico-Santos T (2011). Efficacy of monoterpene perillyl alcohol upon survival rate of patients with recurrent glioblastoma. J. Cancer Res. Clin. Oncol..

[21] Da Fonseca, C. O., Teixeira, R. M., Silva, J. C., Fischer, J. D. S. D. G., Meirelles, O. C., Landeiro, J. A. & Quirico-Santos, T. Long-term outcome in patients with recurrent malignant glioma treated with perillyl alcohol inhalation. *Anticancer Res.* **33**, 5625–5631 (2013). 24324108

[22] Santos JDSS, Diedrich C, Machado CS, Da Fonseca CO, Khalil NM, Marinardes RM (2020). Intranasal administration of perillyl alcohol-loaded nanoemulsion and pharmacokinetic study of its metabolite perillic acid in plasma and brain of rats using UPLC-MS/MS. Biomed. Chromatogr..

[23] Haag JD, Gould MN (1994). Mammary carcinoma regression induced by perillyl alcohol, a hydroxylated analog of limonene. Cancer Chemother. Pharmacol..

[24] Mills JJ, Chari RS, Boyer IJ, Gould MN, Jirtle RL (1995). Induction of apoptosis in liver tumors by the monoterpene perillyl alcohol. Cancer Res..

[25] Belanger JT (1998). Perillyl alcohol: Applications in oncology. Altern. Med. Rev..

[26] Hudes GR, Szarka CE, Adams A, Ranganathan S, McCauley RA, Weiner LM, Langer CJ, Litwin S, Yeslow G, Halberr T, Qian M, Gallo JM (2000). Phase I pharmacokinetic trial of perillyl alcohol (NSC 641066) in patients with refractory solid malignancies. Clin. Cancer Res..

[27] Meadows, S. M., Mulkerin, D., Berlin, J., Bailey, H., Kolesar, J., Warren, D., & Thomas, J. P., Phase II trial of perillyl alcohol in patients with metastatic colorectal cancer. *Int. J. Gastrointest. Cancer* **32**, 125–128, 10.1385/IJGC:32:2-3:125 (2002). 10.1385/IJGC:32:2-3:125 12794248

[28] Ripple GH, Gould MN, Arzoomanian RZ, Alberti D, Feierabend C, Simon K, Binger K, Tutsch KD, Pomplun M, Wahamaki A, Marnocha R, Wilding G, Bailey HH (2000). Phase I clinical and pharmacokinetic study of perillyl alcohol administered four times a day. Clin. Cancer Res..

[29] Cho HY, Wang W, Jhaveri N, Torres S, Tseng J, Leong MN, Lee DJ, Goldkorn A, Xu T, Petasis NA, Louie SG, Schonthal AH, Hofman FM, Chen TC (2012). Perillyl alcohol for the treatment of temozolomide-resistant gliomas. Mol. Cancer Ther..

[30] Da Fonseca, C. O., Khandelia, H., Salazar, M. D., Schonthal, A. H., Meireles, O. C., Quirico-Santos, T. Perillyl alcohol: Dynamic interactions with the lipid bilayer and implications for long-term inhalational chemotherapy for gliomas. *Surg. Neurol. Int.* **7,** 1, 10.4103/2152-7806.173301 (2016). 10.4103/2152-7806.173301 PMC4722523 26862440

[31] Marín-Ramos, N. I., Jhaveri, N., Thein, T. Z., Fayngor, R. A., Chen, T. C., Hofman, F. M. NEO212, a conjugate of temozolomide and perillyl alcohol, blocks the endothelial-to-mesenchymal transition in tumor-associated brain endothelial cells in glioblastoma. *Cancer Lett.* **442,** 170–180, 10.1016/j.canlet.2018.10.034 (2019). 10.1016/j.canlet.2018.10.034 30392789

[32] Wang W, Swenson S, Cho H-Y, Hofman FM, Schönthal AH, Chen TC (2019). Efficient brain targeting and therapeutic intracranial activity of bortezomib through intranasal co-delivery with NEO100 in rodent glioblastoma models. J. Neurosurg..

[33] Wang W, Marín-Ramos NI, He N, Zeng S, Cho H-Y, Swenson SD, Zheng L, Epstein AL, Schönthal AH, Hofman FM, Chen L, Chen TC (2020). NEO100 enables brain delivery of blood-brain barrier-impermeable therapeutics. Neuro Oncol..

[34] Ghersi-Egea JF, Leininger-Muller B, Cecchelli R, Fenstermacher JD (1995). Blood-brain interfaces: Relevance to cerebral drug metabolism. Toxicol. Lett..

[35] Zhang Z, Chen H, Chan KK, Budd T, Ganapathi R (1999). Gas chromatographic-mass spectrometric analysis of perillyl alcohol and metabolites in plasma. J. Chromatogr. B. Biomed. Sci. Appl..

[36] West GB, Brown JH (2005). The origin of allometric scaling laws in biology from genomes to ecosystems: Towards a quantitative unifying theory of biological structure and organization. J. Exp. Biol..

[37] Hua, H-Y., Zhao, Y. X., Liu, L., Ye, Q. X. & Ge, S. W. High-performance liquid chromatographic and pharmacokinetic analyses of an intravenous submicron emulsion of perillyl alcohol in rats. *J. Pharm. Biomed. Anal.* **48 (4),** 1201–1205, 10.1016/j.jpba.2008.08.015 (2008). 10.1016/j.jpba.2008.08.015 18849133

[38] Salazar MD, Da Silva RF, Da Fonseca CO, Lagrota-Candido J, Quirico-Santos T (2014). Intranasal administration of perillyl alcohol activates peripheral and bronchus-associated immune system *in vivo*. Arch. Immunol. Ther. Exp. (Warsz).

[39] Kumar NN, Lochhead JJ, Pizzo ME, Nehra G, Boroumand S, Greene G, Thorne RG (2018). Delivery of immunoglobulin G antibodies to the rat nervous system following intranasal administration: Distribution, dose response and mechanisms of delivery. J. Control. Release..

[40] Thorne RG, Frey WH (2001). Delivery of neurotrophic factors to the central nervous system: Pharmacokinetic considerations. Clin. Pharmacokinet..

[41] Pizzo ME, Wolak DJ, Kumar NN, Brunette E, Brunnquell CL, Hannocks MJ, Abbott NJ, Meyerand ME, Sorokin L, Satnimirovic DB, Thorne RG (2018). Intrathecal antibody distribution in the rat brain: Surface diffusion, perivascular transport and osmotic enhancement of delivery. J. Physiol..

[42] Pajouhesh H, Lenz GR (2005). Medicinal chemical properties of successful CNS drugs. NeuroRx.

[43] Song MS, Wang D, Row KH (2004). Protein binding study of isoflavones by high-performance frontal analysis. Chromatographia.

[44] Boon PJ, van der Boon D, Mulder GJ (2000). Cytotoxicity and biotransformation of the anticancer drug perillyl alcohol in PC12 Cells and in the rat. Toxicol. Appl. Pharmacol..

[45] Miyazawa M, Shindo M, Shimada T (2002). Metabolism of (+)- and (-)-limonenes to respective carveols and perillyl alcohols by CYP2C9 and CYP2C19 in human liver microsomes. Drug Metab. Dispos..

[46] Shimada T, Shindo M, Miyazawa M (2002). Species differences in the metabolism of (+)- and (-)-limonenes and their metabolites, carveols and carvones, by cytochrome P450 enzymes in liver microsomes of mice, rats, guinea pigs, rabbits, dogs, monkeys, and humans. Drug Metab. Pharmacokinet..

[47] Bogdanffy MS, Randall HW, Morgan KT (1987). Biochemical quantitation & histochemical localization of carboxylesterase in the nasal passages of the Fischer-344 rat & B6C3F1 mouse. Toxicol. Appl. Pharmacol..

[48] Bogdanffy MS (1990). Biotransformation enzymes in the rodent nasal mucosa: the value of a histochemical approach. Environ. Heal. Perspect..

[49] Kajiya K, Inaki K, Tanaka M, Haga T, Kataoka H, Touhara K (2001). Molecular bases of odor discrimination: Reconstitution of olfactory receptors that recognize overlapping sets of odorants. J. Neurosci..

[50] Kurosaki M, Terao M, Barzago MM, Bastone A, Bernardinello D, Salmona M, Garattini E (2004). The aldehyde oxidase gene cluster in mice and rats. Aldehyde oxidase homologue 3, a novel member of the molybdoflavoenzyme family with selective expression in the olfactory mucosa. J Biol Chem.

[51] Thornton-Manning JR, Nikula KJ, Hotchkiss JA, Avila KJ, Rohrbacher KD, Ding X, Dahl AR (1997). Nasal cytochrome P450 2A: Identification, regional localization, and metabolic activity toward hexamethylphosphoramide, a known nasal carcinogen. Toxicol. Appl. Pharmacol..

[52] Allali-Hassani A, Martinez SE, Peralba JM, Vaglenova J, Vidal F, Richart C, Farres J, Pares X (1997). Alcohol dehydrogenase of human and rat blood vessels: Role in ethanol metabolism. FEBS Lett..

[53] Galter D, Carmine A, Buervenich S, Duester G, Olson L (2003). Distribution of class I, III and IV alcohol dehydrogenase mRNAs in the adult rat, mouse and human brain. Eur. J. Biochem..

[54] Martinez SE, Vaglenova J, Sabria J, Martinez MC, Farres J, Pares X (2001). Distribution of alcohol dehydrogenase mRNA in the rat central nervous system. Eur. J. Biochem..

[55] Zimatkin S, Lindrost KO (1989). A histochemical study of the distribution of aldehyde dehydrogenase activity in brain structures of rats with genetically different alcohol-related behaviour. Alcohol.

[56] Pierre K, Pellerin L, Debernardi R, Riederer BM, Magistretti PJ (2000). Cell-specific localization of monocarboxylate transporters, MCT1 and MCT2, in the adult mouse brain revealed by double immunohistochemical labeling and confocal microscopy. Neuroscience.

[57] Crowell PL, Lin S, Vedejs E, Gould MN (1992). Identification of metabolites of the antitumor agent d-limonene capable of inhibiting protein isoprenylation and cell growth. Cancer Chemother. Pharmacol..

